# A_2A_ adenosine receptor agonist reduced *MMP8* expression in healthy M2-like macrophages but not in macrophages from ankylosing spondylitis patients

**DOI:** 10.1186/s12891-022-05846-0

**Published:** 2022-10-12

**Authors:** Omid Sadatpour, Mohammad Taha Ebrahimi, Maryam Akhtari, Nooshin Ahmadzadeh, Mahdi Vojdanian, Ahmadreza Jamshidi, Elham Farhadi, Mahdi Mahmoudi

**Affiliations:** 1 Department of Immunology, School of Public Health, Tehran University of Medical Sciences, Tehran, Iran; 2 Rheumatology Research Center, Tehran University of Medical Sciences, Tehran, Iran; 3 Inflammation Research Center, Tehran University of Medical Sciences, Tehran, Iran; 4 Rheumatology Research Center, Tehran University of Medical Sciences, Shariati Hospital, Kargar Ave, P.O. Box: 1411713137, +98-218-822-1449 Tehran, Iran

**Keywords:** Ankylosing spondylitis, Adenosine A_2A_ receptor, Macrophages, Bone morphogenetic protein, Matrix metalloproteinase

## Abstract

**Background:**

Ankylosing spondylitis (AS) is an inflammatory autoimmune disease that mostly affects different joints of the body. Macrophages are the predominant cells that mediate disease progression by secreting several pro-inflammatory mediators. Different receptors are involved in macrophages’ function including the adenosine receptors (AR). Our main objective in this study was to assess the effect of applying A_2A_ adenosine receptor agonist (CGS-21,680) on the gene expression of inflammatory mediators including bone morphogenetic proteins (BMP)-2, 4 and matrix metalloproteinases (MMP)-3, 8, 9, and 13 on the macrophages from AS patients compared to healthy macrophages.

**Methods:**

Monocytes were isolated from the whole blood of 28 individuals (AS patients and healthy controls in a 1:1 ratio). Macrophages were differentiated using macrophage colony-stimulating factor (M-CSF), and flow cytometry was performed to confirm surface markers. CGS-21,680 was used to treat cells that had been differentiated. Using SYBR green real-time PCR, relative gene expression was determined.

**Results:**

Activating A_2A_AR diminished *MMP8* expression in healthy macrophages while it cannot reduce *MMP8* expression in patients’ macrophages. The effect of A_2A_AR activation on the expression of *BMP2* and *MMP9* reached statistical significance neither in healthy macrophages nor in the patients’ group. We also discovered a significant positive connection between *MMP8* expression and patient scores on the Bath ankylosing spondylitis functional index (BASFI).

**Conclusion:**

Due to the disability of A_2A_AR activation in the reduction of *MMP8* expression in patients’ macrophages and the correlation of *MMP8* expression with BASFI index in patients, these results represent defects and dysregulations in the related signaling pathway in patients’ macrophages.

## Introduction

Ankylosing spondylitis (AS) is an autoimmune disorder that mostly affects the sacroiliac joints and the spine. People suffering from this disease typically complain about spinal stiffness and back pain as a result of enthesitis and vertebrae fusion. AS usually starts at an early age and it is believed that one in every two hundred people is influenced by this disease, making it an important health issue [1, 2]. The exact etiology by which the disease is started is still unknown; however, The most important contributors to disease creation are supposed to be hereditary and environmental causes [3]. Diverse environmental factors, infectious diseases, and gut dysbiosis in genetically predisposed people, which is defined by possessing certain human leukocyte antigen (HLA) and non-HLA genes including *HLAB27*, *IL23R*, *ERAP1*, and certain *TLRs* alleles; can cause disease occurrence [1, 4, 5].

Different signaling pathways are involved in autoimmune diseases. One of which is the adenosinergic pathway [6]. To date, available data show that the adenosinergic pathway has a tremendous impact on immunosuppression and aids the body to recover from excessive inflammatory responses. Adenosine is a byproduct of the breakdown of an enzyme cascade, consisting of CD39 (ecto-nucleoside triphosphate diphosphohydrolase 1, E-NTPDase1) breaks down adenosine triphosphate/diphosphate (ATP/ADP) to adenosine monophosphate (AMP) and CD73 (ecto-50-nucleotidase, NT5E) produces adenosine from AMP. Adenosine acts through G-protein-coupled cell-surface receptors which are expressed on a variety of cells, and to date four of them A_1A_R, A_2A_AR, A_2B_AR, and A_3_AR; are recognized. These receptors are known as type 1 purinergic (P1) receptors [6–8]. There is not much information regarding the role of adenosine receptors in AS pathogenesis. However, we have previously reported that macrophages from AS patients expressed elevated levels of A_2A_AR and diminished levels of A_1A_R and A_2B_AR compared to healthy macrophages [9]. Besides, our results demonstrated that A_2A_AR activation results in a reduction in TNF-α production and an increase in *IL23A* expression in AS macrophages [4].

Bone morphogenetic proteins (BMPs) are important members of the transforming growth factor superfamily with multiple vital roles such as embryonic development and osteoblastic differentiation [10]. Of these, BMP-2 is an active and important member which can independently or synergistically with other signaling pathways like the Wnt/β-catenin pathway, enhance osteoblast differentiation and bone formation [10, 11]. Data regarding the level of these proteins in AS patients are incompatible; however, most of them show higher levels of these proteins in patients compared to healthy controls [12, 13].

Matrix metalloproteinases (MMPs) are a family of 23 zinc-dependent enzymes produced by a variety of cells especially immune cells in the event of an inflammatory situation. Inflammatory cytokines such as tumor necrosis factor-alpha (TNF-α), interferon-gamma (IFN-γ), and IL-6 are produced in this environment [14, 15]. These proteins are mostly involved in the degradation and remodeling of extracellular matrices in humans [16]. High levels of MMPs have been seen in inflammatory autoimmune diseases like rheumatoid arthritis (RA). Different studies showed the relation of high levels of MMP-3 and its certain single nucleotide polymorphisms (SNPs) with AS occurrence [16, 17]. Along with the studies showing the relation of MMP-3 and AS, the correlation of bath AS disease activity index (BASDAI) with a group of clustered biomarkers comprising MMP-8, MMP-9, chemokine (C-X-C motif) ligand (CXCL)-8, and hepatocyte growth factor was found [18]. MMP-8 expression is related to inflammatory cytokines and it can have cartilage destructive activities during spondyloarthropathies. One of the SNPs of this enzyme has also been found to be associated with the risk of AS. MMP-9, also called gelatinase B, along with MMP-2 (gelatinase A), are mediators of joint destruction [16, 19].

It is known from previous studies that Inflammatory lesions and the overlaying synovium of spondyloarthritis (SpA) are dominated by macrophages, which are mostly responsible for the breakdown of fibrocartilage [4, 20, 21]. The inflammatory activities of macrophages can be regulated by P1 receptors through binding to adenosine [4]. The expression of proteins responsible for the pathogenesis of AS in macrophages is an important issue to consider for the treatment and control of the disease progression, so we aimed to evaluate the expression level of *MMP3, 8, 9, 13*, and *BMP2*, and *4* in macrophages of AS patients in an untreated situation and after treatment with A_2A_AR agonist (CGS-21,680) as no study has done this up until now.

## Materials and methods

### Study population

With a male/female ratio of 3.6/1 and an average age of 32 ± 10 years, 14 AS patients were chosen. Patients were recruited from the Rheumatology Research Center’s outpatient AS clinic at Shariati Hospital at Tehran University of Medical Sciences, and they all met the modified New York categorization criteria [22]. Patients who had not undergone any disease-modifying drugs or methotrexate were identified for this investigation because certain therapies, such as methotrexate, have a considerable impact on adenosine receptor expression [23]. Simultaneously, the study recruited 14 age and sex-matched healthy persons with a gender and sex distribution similar to AS patients with an average age of 32 ± 96 years. There was no personal or familial history of rheumatic disorders, inflammatory diseases, or psoriasis in the control group. All participants signed a written informed consent form. The Tehran University of Medical Sciences ethical committee accepted this work. (IR.TUMS.DDRI.REC.1399.047).

### Monocyte isolation and macrophage generation

Twenty milliliters of participants’ peripheral blood samples were collected and inserted in tubes containing ethylenediaminetetraacetic acid (EDTA). Within five hours of the time of collection, samples were diluted at 1:2 in phosphate-buffered saline (GIBCO Invitrogen) at a pH of 7.2. Ficoll (Lymphodex, Inno-Train) density gradient centrifugation was used to extract peripheral blood mononuclear cells (PBMCs), which were then washed in PBS. Cell sorter columns were used to separate monocytes after they were treated with MACS CD14 microbeads to undergo positive CD14 selection (all from Miltenyi Biotec). Immunofluorescence labeling of the separated monocytes was done with a phycoerythrin (PE)-conjugated anti-CD14 antibody (BD bioscience) and flow cytometry results revealed a purity of 92–95% [20]. Following that, the isolated CD14 positive monocytes were cultured in the Roswell Park Memorial Institute (RPMI) at a concentration of 500,000 cells per well in 24-well plates. The media contained 2 mM L-glutamine (Biosera), 10% fetal bovine serum (FBS; Gibco BRL), 0.1 mg/ml streptomycin, and 100 U/ml penicillin (Sigma). For seven days, 0.05 µg/ml recombinant macrophage-colony stimulating factor (M-CSF; eBioscience) was added to culture media to convert monocytes into macrophages [24].

### Flow cytometry analysis of macrophage surface markers

The macrophage-specific markers of generated macrophages were examined by flow cytometry using a CyFlow ML flow cytometer (Partec, GmbH, Munster, Germany) and FlowJo software after one weak of monocytes treatment with M-CSF (Tree Star, Ashland, OR, USA) [25]. Cells incubation with fluorescein isothiocyanate (FITC)-conjugated anti-human CD163 and PE-conjugated anti-human CD206, (BD bioscience) or their relative isotype controls was done for half an hour without any light exposure. Analysis of FASC data revealed that 97 and 95% of differentiated macrophages had scavenger receptor CD163 and mannose receptor CD206, respectively [20].

### Real-time quantitative PCR analysis of *MMP* and *BMP* gene expressions

Monocyte-generated macrophages (M2-like) were seeded on RPMI, and half of the specimens were treated with 10 M CGS-21,680 (Sigma), a well-known A2AAR agonist [26]. The High Pure RNA Isolation Kit was utilized to extract total RNA after 24 h (Roche). The complementary DNA (cDNA) was made from the same amount of total RNA using the CellAmp direct RNA prep kit for real-time PCR (Takara bio). StepOnePlus Real-Time PCR equipment (Applied Biosystems) and SYBR green master mix were employed to evaluate the relative expression levels of *MMP3*, *8*, *9*, *13*, *BMP2*, and *4* genes (Ampliqon). Glyceraldehyde-3-phosphate dehydrogenase (*GAPDH*) was used as the endogenous control gene. The primer sequences used for *BMP2* and *4* were from the Harvard Primer Bank. The site https://primer3.ut.ee/ and https://genome.ucsc.edu/ were used to design the *MMP13* primers. The others were selected from previous research. The specific primer sequences and the references were shown in Table 1. For accuracy and specificity, primers were checked using the Basic Local Alignment Search Tool on the US National Center for Biotechnology Information website (HTTP://www.ncbi.nlm.nih.gov/tools/primer-blast/) and https://genome.ucsc.edu/. The mRNA expression level was compared between A_2A_AR agonist (CGS-21,680) treated cells and the untreated group using the comparative CT method (2 ^−ΔΔCT^). It’s worth noting that the cytotoxic potential of CGS-21,680 was tested before the results were interpreted. In this study, the MTT test was utilized to determine the cytotoxicity of CGS-21,680. CGS-21,680 had no harmful effects in this assay, and cell viability was identical to that of untreated cells.

**Table 1:** Primer sequences and product size of the studied genes

Gene name	Sequence	Size (bp)	References
*GAPDH*	F: 5’ GAGTCAACGGATTTGGTCGT 3′ R: 5’ GACAAGCTTCCCGTTCTCAG 3′	185	[9]
*BMP2*	F: 5’ ACTACCAGAAACGAGTGGGAA 3′ R: 5’ GCATCTGTTCTCGGAAAACCT 3′	113	[45]
*BMP4*	F: 5’ ATGATTCCTGGTAACCGAATGC 3′ R: 5’ CCCCGTCTCAGGTATCAAACT 3′	165	[45]
*MMP3*	F: 5’ AGCAAGGACCTCGTTTTCATT 3′ R: 5’ GTCAATCCCTGGAAAGTCTTCA 3′	261	[46]
*MMP8*	F: 5’ TCTGCAAGGTTATCCCAAGG 3′ R: 5’ ACCTGGCTCCATGAATTGTC 3′	154	[47]
*MMP9*	F: 5’ ACCTCGAACTTTGACAGCGA 3′ R: 5’ GTTCAGGGCGAGGACCATAG 3′	220	[48]
*MMP13*	F: 5’ GCAGTCTTTCTTCGGCTTAGAGG 3′ R: 5’TGTATTCACCCACATCAGGAACC 3′	101	ـ

***GAPDH***: Glyceraldehyde-3-phosphate dehydrogenase, ***BMP2***: Bone morphogenetic protein 2; ***MMP***: Matrix metalloproteinase

### Statistical analysis

The Shapiro-Wilk test was used to ensure that all of the variables were normal. To compare non-normally distributed variables, non-parametric methods such as the Mann-Whitney U test were utilized. To compare the levels of mRNA expression in CGS-21,680 treated and untreated cells, the paired sample t-test and Wilcoxon test were performed. The relationship between relative mRNA expression of *BMP2*, *MMP8*, and *MMP9* genes and patient clinical symptoms was investigated using Spearman’s correlation test. Statistical significance was defined as a P value of less than 0.05. SPSS version 22 was used to conduct all statistical analyses, and GraphPad Prism 6 was used to create the graphs.

## Results

### Demographic and clinical characteristics

Table 2 represents the demographic characteristics of enrolled patients and the healthy ones. Clinical scores showing disease severity and activity were also displayed for AS group. According to the report, none of the included patients were on biological or methotrexate therapy.

**Table 2:** Clinical and demographical features of healthy individuals and AS patients

Group	CO individuals (n = 14)	AS patients (n = 14)
Female/Male (%)	3/11 (21/79%)	3/11 (21/79%)
Age, years	32 ± 9.6	32 ± 10
Smoking, %	35	35
ESR, mm/h (SD)	5 (3)	37 (19)
Disease duration, years	-	7.5 ± 6
HLA-B27 positivity, %	-	71
BASMI score (SD)	-	3.7 (2.5)
BASDAI score (SD)	-	6.1 (1.9)
BASFI score (SD)	-	4.6 (2.7)
PGDA score‌ (SD)	-	7 (2.7)
BASG score (SD)	-	7.2 (1.8)
ASQoL score (SD)	-	9.5 (5.6)
Biological agents, %	0	0

AS: Ankylosing spondylitis, CO: Control, ESR: Erythrocyte sedimentation rate, HLA-B27: Human leukocyte antigen (subtypes B*2701–2759), BASMI: Bath Ankylosing Spondylitis Metrology Index, BASFI: Bath Ankylosing Spondylitis Functional Index, BASDAI: Bath Ankylosing Spondylitis Disease Activity Index, BAS-G: Bath Ankylosing Spondylitis Global Score, PGDA: Patient global disease activity, ASQoL: Ankylosing Spondylitis Quality of Life, SD: Standard deviation

### Selected genes mRNA expression

Among selected genes, *MMP3*, *MMP13*, and *BMP4* were not expressed in macrophages from patients and healthy individuals and were excluded from the analysis. M2- like macrophages only expressed *BMP2*, *MMP8*, and *MMP9*.

### ***BMP2*** mRNA expression in M2-like macrophages of AS patients following the A_2A_AR activation

**Fig. 1:**
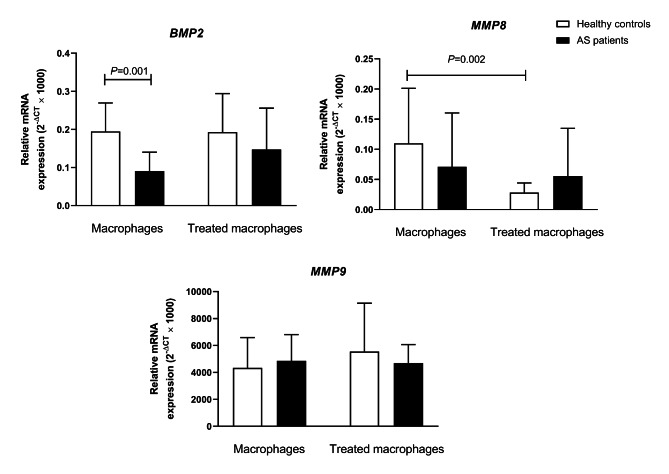
The effect of A_2A_ adenosine receptor agonist (CGS-21,680) on the expression of *BMP2*, *MMP8*, and *MMP9* in monocyte-generated macrophages from AS patients and controls. The data are presented as the mean ± SD

The effect of the A_2A_AR agonist (CGS-21,680) on *BMP2* mRNA expression was determined before and after treating M2-like macrophages of AS patients and healthy controls. Untreated healthy macrophages expressed more *BMP2* than AS macrophages (0.47-fold; P = 0.001, Table 3). CGS-21,680 raised *BMP2* mRNA expression in AS patients’ macrophages by 1.62-fold, which was not statistically significant (P = 0.167), and it had no effect on *BMP2* mRNA expression in healthy people’s macrophages (Fig. 1).

**Table 3:** The mRNA expression of *BMP2*, *MMP8*, and *MMP9* in monocyte-derived (M2-like) macrophages from AS patients and healthy controls

	Relative gene expression			
	**Healthy macrophages**	**AS macrophages**	**Mean fold changes (95% CI)**	***P*** **-value**
***BMP2***	0.19 ± 0.07	0.09 ± 0.05	0.47 (0.31, 0.7)	**0.001**
***MMP8***	0.11 ± 0.09	0.07 ± 0.09	0.64 (0.25–1.66)	0.311
***MMP9***	4333.5 ± 2247.9	4860.4 ± 1943.2	1.12 (0.77, 1.63)	0.529

***BMP***: Bone morphogenetic protein; ***MMP***: Matrix metalloproteinase

### *MMP8 mRNA expression in AS patients’ M2-like macrophages after A*a_*2A*_*AR activation*

The expression of *MMP8* did not differ between untreated healthy macrophages and AS ones (Table 3). The CGS-21,680 decreased the mRNA expression level of *MMP8* in monocyte-derived macrophages of healthy individuals by 0.26-fold (P = 0.002). On the other hand, the decrease noticed in AS patients was not statistically significant (0.78-fold; P = 0.122) (Fig. 1; Table 4).

**Table 4:** The mRNA expression of *BMP2*, *MMP8*, and *MMP9* in monocyte-derived (M2-like) macrophages from AS patients and healthy controls before and after CGS-21,680 treatment

	Relative gene expression			
	**Healthy macrophages**	**Treated healthy macrophages**	**Mean fold changes (95% CI)**	***P*** **-value**
***BMP2***	0.19 ± 0.07	0.19 ± 0.10	0.99 (0.77, 1.26)	0.974
***MMP8***	0.11 ± 0.09	0.03 ± 0.01	0.26 (0.19, 0.36)	**0.002**
***MMP9***	4333.5 ± 2247.9	5556.2 ± 3580.8	1.28 (0.98, 1.68)	0.101
	**AS macrophages**	**Treated AS macrophages**	**Mean fold changes (95% CI)**	***P*** **-value**
***BMP2***	0.09 ± 0.05	0.15 ± 0.11	1.62 (0.96, 2.72)	0.167
***MMP8***	0.07 ± 0.09	0.05 ± 0.08	0.78 (0.45, 1.34)	0.122
***MMP9***	4860.4 ± 1943.2	4696.05 ± 1372.1	0.97 (0.84, 1.11)	0.729

***BMP***: Bone morphogenetic protein; ***MMP***: Matrix metalloproteinase

### *MMP9 mRNA expression in AS patients’ M2-like macrophages after A*a_*2A*_*AR activation*

We did not find any significant differences in the expression of *MMP9* between AS and healthy macrophages (Table 3). Besides, the A_2A_AR agonist couldn’t significantly change *MMP9* mRNA expression level in macrophages from AS patients and healthy persons. (Fig. 1; Table 4).

### Correlation between the relative mRNA expression with clinical manifestations of AS patients

The link between the expression level of studied genes with clinical manifestations of AS patients was also evaluated. A significant positive correlation between *MMP8* and BASFI level (Bath Ankylosing Spondylitis Functional Index) was also seen in AS patients (P = 0.034) (Table 5).

**Table 5:** Matrix of Spearman’s correlation coefficient between the mRNA expressions of *BMP2*, *MMP8*, and *MMP9* genes and clinical manifestations of patients

	***ESR***	***BASMI***	***BASFI***	***BASDAI***	***BASG***	***PGDA***	***ASQol***
***BMP2***	− 0.133	0.049	0.132	− 0.066	− 0.025	− 0.051	0.037
***MMP8***	0.220	0.148	0.639*	0.498	− 0.042	0.395	0.458
***MMP9***	0.082	− 0.114	− 0.025	− 0.006	0.014	0.183	− 0.150

***BMP***: Bone morphogenetic protein, ***MMP***: Matrix metalloproteinase, ESR: Erythrocyte sedimentation rate, BASMI: Bath Ankylosing Spondylitis Metrology Index, BASFI: Bath Ankylosing Spondylitis Functional Index, BASDAI: Bath Ankylosing Spondylitis Disease Activity Index, BASG: Bath Ankylosing Spondylitis Global Score, PGDA: Patient global disease activity, ASQoL: Ankylosing Spondylitis Quality of Life, SD: Standard deviation. * p < 0.05; ** p < 0.01; *** p < 0.001

## Discussion

Regulating inflammation in autoimmune diseases like SpA is very important in ameliorating disease signs. Different inflammatory molecules take part in disease progression like matrix metalloproteinases which contribute to bone and joint degeneration to a great extent [15].

Matrix metalloproteinases are one of many inflammatory mediators in AS patients. They are mostly involved in bone and cartilage degeneration; and are considered a good therapeutic target, especially in a group of patients that don’t respond to TNF therapies who are called non-responders to TNF inhibitor therapies [15, 27]. We investigated the gene expression levels of four matrix metalloproteinases: MMP-3, MMP-8, MMP-9, and MMP-13, which have been linked to pro-inflammatory roles in disease pathogenesis in earlier investigations. We did not detect *MMP3* and *MMP13* expression in isolated monocyte-derived macrophages. Sames as our results, Huang et al. did not detect *MMP13* expression in unstimulated differentiated macrophages and *MMP3* was expressed at a low level in them [28]. It is also demonstrated that *MMP3* expression is induced in primary macrophages only after LPS stimulation [29].

MMP-8 which is predominantly produced by activated neutrophils and therefore called neutrophil collagenase, besides AS plays important pathogenic roles in other inflammatory autoimmune diseases like multiple sclerosis (MS) and RA [30, 31]. The relation between certain SNPs of *MMP8* and AS occurrence has also been shown in previous research [19]. In addition, the serum level of MMP-8 has been shown to be higher in AS patients and associated with disease activity [18, 32]. Here, in this study, although we didn’t see a significant difference in *MMP8* gene expression between macrophages from AS patients and healthy controls, we saw a positive correlation between *MMP8* expression level and BASFI index in patients. This finding is showing that higher levels of *MMP8* expression in macrophages may contribute to the inflammatory process in patients and could influence patients’ ability to cope with everyday life [18]. Applying A_2A_AR agonist, as an anti-inflammatory agent, on macrophages significantly reduced *MMP8* expression in healthy controls but didn’t have this significant reduction in AS patients. Previously we have reported that AS patients’ macrophages express more A_2A_AR than healthy people’s macrophages [19]. The fact that activation of A_2A_AR cannot diminish the *MMP8* expression in patients’ macrophages may be due to defects or dysregulations in the related signaling pathway in AS patients.

Just like MMP-8, MMP-9 is another important enzyme that plays a part in multiple autoimmune diseases [33, 34]. In AS specifically, MMP-9 has cartilage destructive activities through degrading type IV collagen in extracellular matrices and facilitating lymphocytes’ entrance into sites of inflammation [34]. Higher amounts of this enzyme have been detected in the serum of AS patients along with MMP-8 and CXCL-8 and their relevance to disease activity status has been shown too [18]. Here, we didn’t see any difference in *MMP9* gene expression between macrophages from healthy subjects and AS patients. Previously it was demonstrated that activation of the A_2A_AR receptor can diminish *MMP9* expression in neutrophils [35]. Besides Chen et al. found that A_2B_AR activation reduces *MMP9* expression in macrophages depending on TNF-α [36]. Previously we reported that A_2A_AR agonist can reduce TNF-α production in macrophages from AS patients [4]. It has been also demonstrated that TNF-α upregulates MMP9 expression [37, 38]. however, here we saw that activation of A_2A_AR receptor couldn’t affect the baseline expression level of *MMP9* in macrophages from both healthy and AS groups. Nevertheless, we did not detect MMP-9 protein expression level and further study is required.

Besides chronic inflammation, AS is characterized by the bone formation and ankyloses of the sacroiliac joints while BMPs play a pivotal role in this process [39]. BMPs are a group of transforming growth factors and cytokines that can induce bone formation [40]. Inflammatory conditions and pro-inflammatory cytokines can induce BMPs expression. Previously demonstrated that macrophages participate in osteoinduction and early steps of bone formation by producing BMPs molecules [41]. It is demonstrated that M2 type of macrophages that participate in wound healing express an upregulated level of BMP-2 compared to M1 macrophages [41, 42]. Due to the role of macrophages in AS pathogenesis, here we investigated the expression of BMP2 in M2-like macrophages of patients and controls. In a meta-analysis by Yang et al., it is demonstrated that serum BMP-2 level is significantly higher in patients compared to controls [10]. Imbalances between different molecules in the BMP-2 signaling pathway also play a role in inducing bone formation and disease progression and participate in abnormal osteogenic differentiation of mesenchymal stem cells [39, 43]. It has also been shown by different studies that higher BMP-2 levels in AS patients are usually accompanied by higher BASDAI and c-reactive protein (CRP) levels [10, 44]. In the present study, we did not measure the level of BMP-2 in the serum of AS patients, and for the first time, we analyzed *BMP2* gene expression in macrophages of patients and healthy people. We saw higher *BMP2* expression in the macrophages of healthy subjects than in AS patients. The decreased expression of *BMP2* in patients’ macrophages may be due to the negative feedback loop to suppress excessive bone formation in patients. Activation of A_2A_AR didn’t affect *BMP2* levels in macrophages from patients and controls. We also did not see any association between the level of *BMP2* gene expression in macrophages and the level of patients’ clinical manifestations.

## Conclusion

In conclusion, the present study shows that activating A_2A_AR on macrophages cannot reduce *MMP8* expression in patients’ macrophages while it diminished *MMP8* expression in healthy macrophages. These results represent a dysregulation in the related signaling pathway in AS patients. Moreover, we found a significant positive correlation between *MMP8* gene expression level and BASFI score in patients, telling us that higher *MMP8* expression can be associated with higher BASFI and patients’ incapacitation. The effect of A_2A_AR activation on the expression of BMP2 and MMP9 did not reach statistical significance.

However, the small sample size and healthy controls as comparators are the limitations of the current study. Besides, other relevant gene expressions, protein levels in the supernatant, and the entire downstream pathway were not assessed in our study. Therefore, further studies with bigger sample sizes on different genes and signaling pathways in macrophages of AS patients can be useful to investigate and determine the exact effect of A_2A_AR on the pathogenesis of the disease.

## Data Availability

Data are available on request from the corresponding authors.

## Abbreviations

ADP: Adenosine diphosphate
AMP: Adenosine monophosphate
AR: Adenosine receptor
AS: Ankylosing spondylitis.
ATP: Adenosine triphosphate
BASDAI: Bath ankylosing spondylitis activity index
BASFI: Bath Ankylosing Spondylitis Functional Index
BMP: Bone morphogenetic protein
CD39: Ecto-nucleoside triphosphate diphosphohydrolase 1, E-NTPDase1
CD73: Ecto-50-nucleotidase, NT5E
CRP: C-reactive protein
CXCL: Chemokine (C-X-C motif) ligand
EDTA: Ethylenediaminetetraacetic acid
GAPDH: Glyceraldehyde-3-phosphate dehydrogenase
IFN-γ: Interferon-gamma
M-CSF: Macrophage colony-stimulating factor
MMP: Matrix metalloproteinases
MS: Multiple sclerosis
PBMC: Peripheral blood mononuclear cell
RA: Rheumatoid arthritis
RPMI: Roswell Park Memorial Institute
SNP: Single nucleotide polymorphism
SPA: Spondyloarthritis
TNF-α: Tumor necrosis factor-alpha
